# Therapeutic Applications of Engineered Mesenchymal Stromal Cells for Enhanced Angiogenesis in Cardiac and Cerebral Ischemia

**DOI:** 10.1007/s12015-024-10787-3

**Published:** 2024-09-21

**Authors:** Madhavi Hegde, Abhishek Kumar Singh, Suresh Kannan, Udaykumar Kolkundkar, Raviraja N. Seetharam

**Affiliations:** 1 Manipal Centre for Biotherapeutics Research, Manipal Academy of Higher Education, Karnataka, Manipal, 576 104 India; 2 Stempeutics Research Pvt. Ltd., 3rd Floor, Manipal Hospitals Whitefield #143, EPIP Industrial Area, ITPL Main Road, Bangalore, 560 048 India

**Keywords:** Angiogenesis, Gene modification, Ischemia, Mesenchymal stromal cells, Secretome

## Abstract

Ischemic diseases are characterized by obstruction of blood flow to the respective organs, of which ischemia of the heart and brain are the most prominent manifestations with shared pathophysiological mechanisms and risk factors. While most revascularization therapies aim to restore blood flow, this can be challenging due to the limited therapeutic window available for treatment approaches. For a very long time, mesenchymal stromal cells have been used to treat cerebral and cardiac ischemia. However, their application is restricted either by inefficient mode of delivery or the low cell survival rates following implantation into the ischemic microenvironment. Nonetheless, several studies are currently focusing on using of mesenchymal stromal cells engineered to overexpress therapeutic genes as a cell-based gene therapy to restore angiogenesis. This review delves into the utilization of MSCs for angiogenesis and the applications of engineered MSCs for the treatment of cardiac and cerebral ischemia. Moreover, the safety issues related to the genetic modification of MSCs have also been discussed.

**Graphical Abstract:**

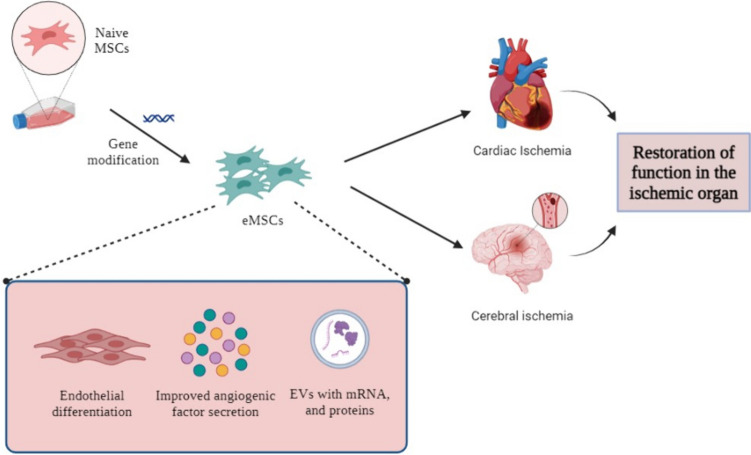

## Introduction

Ischemia is caused by the failure of vital organs to receive enough blood and oxygen [108, 129]. Out of these, the most prevalent and fatal types are the ones affecting the heart and brain [117]. According to the Indo-US Collaborative Stroke Project, hypertension (60.8%) and diabetes mellitus (35.7%) were common risk factors among 2,066 ischemic stroke patients, with cardiac causes accounting for 24.9% of stroke cases [121]. Furthermore, a study that investigated patients who experienced cerebrovascular accidents discovered that 14.78% of them had an ST-elevation myocardial infarction, emphasizing the connection between cerebral ischemia and cardiac events [88]. Overall, these results highlight the critical need for focused interventions to address India's high rate of ischemic conditions, especially in urban populations where the burden is higher [112, 119]. The standard treatment for tissue ischemia involves intravenous (IV) thrombolysis with recombinant tissue plasminogen activator (rtPA) and mechanical thrombectomy, that are associated with a limited therapeutic window, patient eligibility, as well as hemorrhagic complications [90]. Pharmacological interventions or revascularization are the cornerstones of the current ischemia treatment paradigm. However, it is possible that the nearby tissues may not regain their function fully, which poses the need to find better alternative treatments [54]. Cell therapy using mesenchymal stromal cells (MSCs) can be utilized for such no-option patients, who have limited revascularization possibilities [108].

MSCs are multilineage stromal cells that originate from progenitor cells that have undergone partial differentiation and are often recovered via density gradient centrifugation or immunophenotyping [48, 124]. The benefits of employing MSCs for therapies include their simple extraction and expansion procedures, their ability to differentiate into adipocytes, osteocytes, chondrocytes, and many other cell types, their capacity to localize to the site of inflammation or injury, and their ease in transfection, which enables in vitro genetic alterations. [43]. The generation of extracellular vesicles, immune regulation, and the ability to achieve disease-specific therapeutic results are just a few of the important biological functions carried out by MSCs that could be augmented through gene modification [130]. These gene modified MSCs, hereby referred to as engineered MSCs (eMSCs), are programmed to overexpress or inhibit therapeutic proteins and growth factors, which improves their capacity for paracrine action, differentiation, proliferation, and survival under hypoxic conditions [43, 95, 101, 130]. The advantage of this strategy is that by integrating MSC-mediated cell therapy along with genetic modification, MSCs can be used as delivery platforms for therapeutic factors [5].

Angiogenesis refers to the process of formation of new vasculature by branching from pre-existing “parental” blood vessels, wherein destabilization of vasculature, angiogenic sprouting, formation of lumen, and restabilization of vasculature occurs in a sequential manner [9, 55]. Since blood vessels are necessary for the movement of oxygen and other nutrients, as well as for the excretion of carbon dioxide and metabolic wastes from the body, impairment of angiogenesis is linked to a variety of disorders [55]. Among the existing angiogenic therapies, cell therapy utilizing stem cells is a promising avenue for enhancing the development of de novo microvessels in ischemic areas [63, 109]. Previous findings in stem cell research have shown that MSCs are essential for stimulating angiogenesis, particularly for the treatment of ischemic diseases, through differentiation, communication with endothelial cells (ECs), and paracrine secretion of pro-angiogenic factors [45, 85, 93, 106, 136]. However, their low survival rate in target tissues and limited ability to transdifferentiate in vivo after transplantation suggests paracrine activity as the major therapeutic mechanism [83, 106].

In this review, we present a general overview of how MSCs and eMSCs contribute to the stimulation of angiogenesis. The review's opening section provides an outline of how MSCs facilitate the formation of blood vessels through their secretome. Also, the interaction between MSCs and different cell types and their mode of operation to promote angiogenesis are described. The emergence of eMSCs, along with their benefits and drawbacks compared to cell therapy employing MSCs, is then briefly discussed. The second part of the paper focuses on new findings of growth factor and chemokine overexpression for therapeutic angiogenesis and their usage in clinical settings to overcome ischemia of the heart and brain. The need for eMSCs and the prospects for translating these therapies to the market are covered in the last section.

## The Role of MSCs in Angiogenesis: Molecular Mechanism

Angiogenesis is a tightly regulated process that involves interactions between ECs and their surrounding environment and is crucial for tissue repair, bone regeneration, and wound healing [65, 78]. It is also essential for the transfer of hormones and immune cells to target areas in addition to providing nutrients and waste elimination [104]. Cytokines were originally used as the basis for therapeutic angiogenesis, but the absence of potent therapies has moved attention to stem cell transplantation [104]. MSCs are regarded as therapies for vascular regeneration due to their ability to differentiate into smooth muscle cells (SMCs) and ECs, as well as secrete trophic substances. The advantage of employing MSCs over traditional methods is that MSC-differentiated vascular cell lineages can directly contribute to blood flow restoration through the development of new blood vessels, and the trophic factors boost the angiogenic capability that already exists in vivo [37]. Unlike endothelial progenitors, MSCs might not show diminished angiogenic potential when compared to healthy volunteers, making them a viable treatment alternative [35, 116].

One of the key processes behind the MSCs' angiogenic capacity is their paracrine activity, whereby they produce a variety of substances known collectively as the secretome [80]. It consists of two fundamental components, which are: cytokines, chemokines, and growth factors that promote in vitro and in vivo angiogenesis; and extracellular vesicles that can deliver angiogenic molecules, genetic materials, and lipids [6, 57, 124]. MSCs from diverse tissue sources release growth factors in varied amounts, which influences the secretome. Furthermore, the secretome profile might be affected by the priming or preconditioning that MSCs receive during cell culture before the collection of conditioned media [22, 82, 125]. The major approaches that are used to engineer the MSC secretome include hypoxia [26, 146], exposure of MSC culture media to certain growth factors or bioactive agents [58, 60], and modulating the cell-to-cell and cell-to-extracellular matrix (ECM) interactions [13, 96].

Numerous angiogenic factors, including vascular endothelial growth factor (VEGF), hepatocyte growth factor (HGF), transforming growth factor beta (TGF-β), platelet-derived growth factor (PDGF), placental growth factor (PlGF), Ang-1, and others, are secreted by MSCs in their extracellular space. These factors promote angiogenic behavior and EC migration as observed in MSCs from many different tissues [8, 21, 36, 59, 102, 122]. A study found that the combination of several growth factors, namely VEGF, HGF, TGF-β1, interleukin-6 (IL-6), and monocyte chemotactic protein-1 (MCP-1), secreted in MSC-derived conditioned media, promoted angiogenic activity of ECs in vitro and increased the blood flow and angiogenesis rate in vivo in a mouse model of hind limb ischemia [63]. In vitro, MSCs can also induce ECs to break down the surrounding ECM. Further, when ECs are cultivated in three-dimensional aggregates as opposed to monolayers, the conditioned medium from BM-MSCs exhibits improved EC proliferation, invasion, and migration. The increased levels of VEGF, angiogenin, fibroblast growth factor 2 (FGF-2), and IL-11 produced could be the cause of this finding [102].

The MSC-EC interaction is another important part of the angiogenic effect shown by MSC transplantation. When MSCs are co-implanted subcutaneously with endothelial progenitor cells (EPCs) in Matrigel into immunodeficient mice, they are able to form anastomes and generate a complex network of microvascular beds containing erythrocytes within a week of transplantation [86]. Additionally, it was noted that MSCs were located next to the luminal aspect of the vessels, indicating that they were serving as perivascular support for the developing blood vessels, whereas EPCs were confined to the lumen [10, 86]. While the use of EPCs alone results in the formation of sparse networks in contrast to limited vascularization by MSCs alone, it is observed that a combination of both the cell types resulted in optimum blood networks [1]. Furthermore, it has been demonstrated that hybrid spheroids containing MSCs and endothelial colony-forming cells (ECFCs) greatly increase the engraftment and survival of ECFCs, improving therapeutic angiogenesis in models of peripheral artery disease [118]. Additionally, co-culture systems demonstrate that MSCs can influence ECs by paracrine signaling and direct contact, which encourages tube formation and stabilizes vascular structures [104]. The complex role of MSCs in angiogenesis is highlighted by the interaction between ECs and MSCs that involves pathways like uPA-uPAR and Notch signaling. This interaction further promotes the development of endothelial tubular networks [7]. Therefore, MSC-EC interactions are essential for in vivo angiogenic processes to be successful. However, current research also emphasizes how mitochondria improve EC bioenergetics independently of MSC vascular support [79, 115]. While MSCs transfer mitochondria to enhance EC-mediated angiogenesis, there is a comparable therapeutic effect when mitochondria are transferred via tunneling nanotubes, which can also remove the need for MSCs. This is an emerging field that requires further investigation.

Extracellular vesicles (EVs), that possess angiogenic potential in addition to other benefits, make up a sizable fraction of the paracrine components produced by MSCs [32]. They are small, 30–150 nm membrane-bound vesicles that are released by a variety of cells and are generated when the endosomal membrane bulges inward, thereby forming multi-vesicular bodies that fuse with the plasma membrane [110, 126]. They play significant roles in cellular communication by transporting soluble proteins and genetic material like mRNAs and microRNAs (miRNAs) [32, 78, 110]. Many studies have reported that MSC-derived exosomes support angiogenesis by promoting EC proliferation, migration, and tube formation in vitro [34, 47, 61, 66, 148]. Exosomes derived from AD-MSCs have been shown to be taken up by the ECs and aid in the angiogenesis process through transfer of miR-125a.

## Engineered MSCs for Angiogenesis Applications

The biological characteristics and clinical efficiency of MSCs are influenced by several variables, including the dosage of implanted cells, their purpose, their use for a particular clinical indication, and the time of administration [29, 84, 134, 151]. Several preclinical in vivo animal investigations have demonstrated that anoikis, inflammation, and ischemia impair MSCs' ability to engraft as well as their survival and homing in target tissues, which hinders their therapeutic efficacy [95]. The curative properties of MSCs can be increased by a variety of methods, including co-transplantation with graft materials, priming or preconditioning, and gene modification [87]. Preconditioning involves subjecting MSCs to hypoxia, chemical and pharmaceutical compounds, growth factors, cytokines, etc. before their application. Several studies have shown that MSCs’ paracrine capacity is boosted by hypoxia preconditioning [12, 67]. While pretreated MSCs offer better survival, differentiation, homing and paracrine effects, further research on optimization of factors required for priming and their underlying mechanisms is required for better understanding [46].

On the other hand, gene modification results in eMSCs that are altered to express specific exogenous genes that strengthen tissue homing to the target sites and angiogenesis, promoting MSC engraftment, function, and in addition delivering the therapeutic gene outcomes for tissue-specific healing [74, 76, 95, 111]). Additionally, it is more efficient and bioavailable than merely injecting therapeutic proteins [24]. These eMSCs have a multitude of applications that include: 1. Generating MSCs with improved regenerative and immunomodulatory function that are more disease-specific; and 2. Using MSCs as the carrier for delivering therapeutic genes and/or for secreting therapeutic proteins [130]. The fundamental procedure for genetically altering MSCs to create eMSCs entails extracting MSCs from patients, packaging the gene of interest into a vector that will deliver it to the target MSCs, and reintroducing eMSCs into the same patients to carry out the intended function [158].

There have been several approaches utilized to create eMSCs, where the gene of interest is inserted into MSCs using both viral and non-viral vectors. Viral vectors like lentivirus/retrovirus, adenovirus, and adeno-associated virus are used in around 70% of gene therapy-based clinical trials worldwide [114]. This is mostly because of their better tissue tropism, higher transduction efficiencies, and sustained transgenic expression. They are also linked to a low cargo carrying capacity, high immunogenicity, off-target effect that causes insertional mutagenesis, and a high cost of manufacture [23, 62, 95, 130, 144]. Non-viral vector-mediated delivery using plasmid DNA, nanoparticles, liposomes, and DNA minicircles has a comparatively lower immunogenicity, superior loading capacity, and is simpler to construct and target tissues, along with the ease of scale-up during manufacturing. But, their usage has been constrained by their reduced efficiency and transient transgene expression [74, 97, 101, 144]. Recently, therapeutic genes have also been overexpressed in MSCs through the use of CRISPR-Cas9 [28]. Moreover, it has been shown that the use of ribonucleoprotein (RNP) delivery systems improves therapeutic outcomes and decreases cytotoxicity while increasing the efficacy of genome editing in MSCs [40]. The therapeutic response ultimately determines the best vector system to be employed for cell-based gene therapy, and this decision will have a significant impact on how well the transplanted cells perform [130].

## Therapeutic Effect of eMSCs for Ischemia

**Fig. 1:**
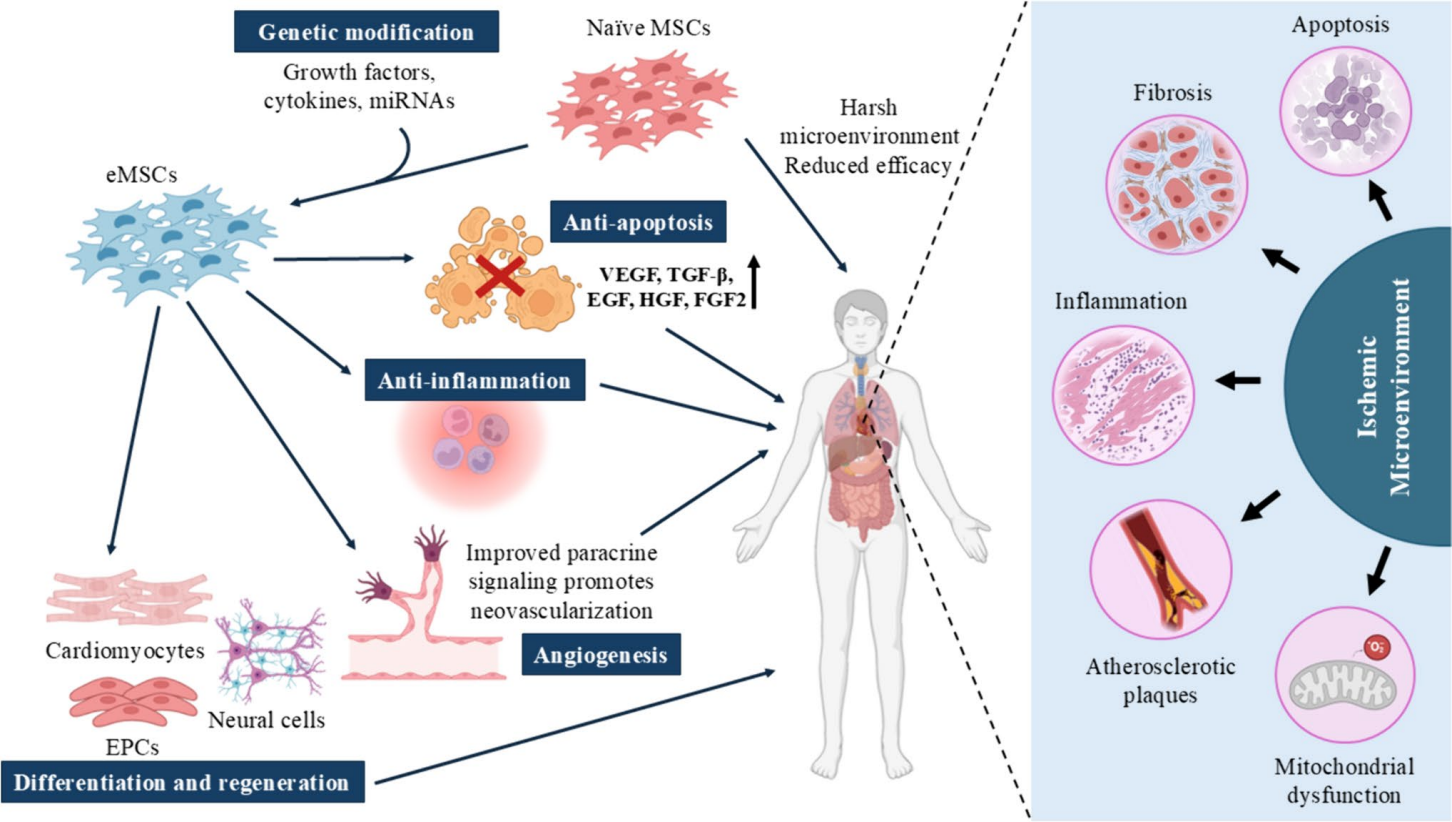
Enhanced Therapeutic Effects of Engineered MSCs (eMSCs) in Ischemia. Naïve MSCs generally encounter poor survival and apoptosis in the harsh ischemic environments. Genetic modifications of MSCs enhance their angiogenesis, survival, and regenerative abilities, improving ischemic repair. (MSCs—mesenchymal stromal cells, eMSCs – engineered mesenchymal stromal cells; VEGF – Vascular endothelial growth factor; TGF-β – Transforming Growth Factor Β; EGF – Epidermal Growth Factor; HGF – Hepatocyte Growth Factor, FGF2—Fibroblast Growth Factor 2) (Created with Biorender.com and M.S. Powerpoint)

Reduced blood flow to the organs because of a blood artery obstruction is known as ischemia [108]. MSCs can help restore tissue homeostasis and repair ischemia by promoting angiogenesis. Their therapeutic potential is mediated by trilineage differentiation in conjunction with the release of bioactive factors and exosomes into the extracellular medium [49]. Nowadays, a great deal of ischemic conditions are well-characterized, and MSC-based therapies are available for most of them. These ailments, which impair the operation of vital organs including the heart, brain, lungs, extremities, intestines, and so on, include ischemic heart disease, cerebral ischemia, critical limb ischemia or peripheral artery disease, retinal ischemia, and many more [25]. A significant danger of cardiovascular disorders, which may cause the patient's death, is posed by many of these ailments [108]. Although reperfusion therapy, pharmacological interventions and surgeries could be used as the primary resort to counteract ischemia, it may restore the function of arteries. However, it is possible that the surrounding tissues may not regain their function or they will not be able to promote regeneration [54]. For no-option patients for whom revascularization is not feasible, cell therapy using MSCs alone or in combination with gene therapy could be a viable option [108]. The next section will cover the application of eMSCs to restore angiogenesis as well as their mode of action in cardiac and cerebral ischemia. The major therapeutic effects driven by eMSCs against tissue ischemia have been depicted in Fig. 1.

### Cardiac Ischemia

Ischemic heart diseases (IHDs), most importantly myocardial infarction (MI), have emerged as a major global cause of death [147]. Given that inadequate angiogenesis is the root cause of most of them, restoring it may be necessary for most therapeutic strategies [120]. The associated rate of mortality remains the main issue in many countries, despite advancements in treatment alternatives like bypass surgery or the implantation of stents, in addition to traditional medications and therapies that have reduced it [41]. Due to the loss of viable myocardium and subsequent tissue remodeling, it could result in end-stage heart failure. For individuals with end-stage IHDs, heart transplantation is the sole option available because the cardiomyocytes that are terminally differentiated have no further potential for regeneration [147]. Cell therapy using MSC transplantation has been explored by numerous scientists to provide a regenerative therapy for cardiac repair, and they have also obtained positive outcomes. However, the buildup of surplus branched chain amino acids after ischemia further decreases the cardioprotective benefits of implanted MSCs, indicating that the ischemic heart's metabolic alterations play a significant role in determining the MSCs' ultimate fate and effectiveness [152]. Therefore, studies that use eMSCs that overexpress various genes are now being conducted to circumvent the drawbacks of MSC-based treatment.

Many studies have used angiogenic factors such as VEGF, FGF2, and stromal cell derived factor 1α (SDF1α) overexpressed cells to overcome ischemia [31]. VEGF is the most crucial factor of them all, known to regulate EC proliferation, migration, and tube formation [100]. Zhu et al. created MSCs that overexpressed VEGF and implanted them into a mouse model of MI. These cells expressed VEGF for a prolonged period, peaking on day 2, and showed that they could promote the growth of ECs. They also secreted VEGF at the ischemic regions, increasing MSC survival and inducing neovascularization, leading to improved cardiac function [157]. Furthermore, in rat models of MI, the hypoxia-inducible VEGF-overexpressed MSCs generated by Moon et al. showed improved LV remodeling and vascular formation in addition to having a higher cell viability in vitro [94]. HGF is another angiogenic factor required for EC growth, that has also demonstrated potent anti-inflammatory and anti-apoptotic effects [52, 100, 154]. In a mouse model of MI, Zhao et al. investigated the potential of HGF-engineered UC-MSCs for cardioprotection. Hypoxic neonatal cardiomyocytes were treated with the conditioned medium obtained from UC-MSCs and HGF-UC-MSCs under hypoxic conditions, wherein the engineered cells exhibited reduced apoptosis. In comparison to the control cells, the conditioned media from these cells also revealed higher concentrations of HGF, VEGF, Epidermal Growth Factor (EGF), and basic Fibroblast Growth Factor (bFGF). Proving the therapeutic efficacy of HGF-UC-MSCs over UC-MSCs, there appeared to be an increased angiogenesis and enhanced cardiomyocyte proliferation when both types of cells were injected into the ischemic myocardium [154]. Similar finding of elevated angiogenesis was also reported by Wang et al., where HGF was overexpressed in BM-MSCs [135].

Hypoxia-induced factor 1α (HIF1α) is another angiogenic factor that has anti-remodelling and anti-apoptotic benefits. When this gene was integrated into porcine MSCs with a non-viral circular minicircle DNA vector and injected endomyocardially into porcine model of IHD, the result was reduced infarct size and improved cardiac function supported by enhanced angiogenesis [30]. Shortly after transplantation, there was also an increase in the expression levels of several angiogenic genes and miRNAs. This study highlights the translational potential of eMSCS as it uses a non-viral approach to target IHD. However, the authors have acknowledged certain limitations such as the use of a smaller number of animals, brief follow-up period, and need to ascertain long-term effects and biodistribution. Similar results were also observed after myocardial injection of endothelial nitric oxide synthases (eNOS) overexpressed MSCs into a rat model of acute MI [18]. Integrin-linked kinase (ILK) is a gene expressed in the heart, that is implicated in angiogenesis and myocardial contraction [113]. When MSCs with ILK engineering were injected into an ischemic heart, as opposed to MSC control and MSCs with ILK silenced, there was a greater increase in angiogenesis and MSC survival, as reported by Zeng et al. This was linked to better cardiac remodelling and decreased infarct size and fibrosis [150]. Chen et al., engineered MSCs with miR-126, an EC specific miRNA that promotes neoangiogenesis and maintains vascular integrity post MI [27]. After being implanted intramyocardially into mice modelling MI, these cells exhibited prolonged survival and secreted miR-126 for a minimum of six weeks. Additionally, they outperformed naïve MSCs in terms of cardiac function, arteriogenesis, and vessel density. The primary outcome of this work indicated a correlation between elevated angiomyogenesis and AKT/ERK-related pathway activation [15].

There have been reports of several studies where genetic modification with several supporting genes have resulted in a better angiogenesis in ischemic hearts. For example, Tsubokawa et al., employed BM-MSCs transiently overexpressed with Heme Oxygenase-1 (HO-1), an anti-inflammatory and antioxidant protein, for ischemic myocardial injury. These overexpressed cells (^HO−1^MSCs) were resistant to cell death and apoptosis under serum deprivation, hypoxia or H_2_O_2_ induced oxidative stress, and secreted a 2.1-fold greater amount of VEGF than normal MSCs. Additionally, they witnessed lower VEGF levels in BM-MSCs and ^HO−1^MSCs that had received a PI3/Akt pathway inhibitor pretreatment. The transplanted rat infarction model exhibited an increase in capillary density, a decrease in the number of cells undergoing apoptosis, and a reduction in the size of the infarct in vivo [128]. It has also been demonstrated that MSCs with the stromal-derived factor-1 alpha (SDF-1α) gene modified can effectively induce angiogenesis in vivo, resulting in increased capillary density, thicker left ventricular (LV) wall, and smaller infarcts and fibrosis [123]. In another study, the overexpression of glycogen synthase kinase-3β (GSK-3β) in MSCs was linked to improved cardiac function. This was accompanied by increased production of VEGFA, which improved paracrine function, and increase in the capillary density. Even after being knocked down with VEGFA, these cells were still able to shrink MI and enhance LV function, but they did not increase vessel density or survival. This demonstrates that MSCs have the ability to treat MI both through VEGFA-dependent and -independent pathways [19].

Forkhead box C1 (FoxC1) is a gene that maintains the stem cell niche found in hair follicles and regulates stem cells' ability to regenerate tissue. The injection of genetically modified MSCs expressing the FoxC-1 gene resulted in increased secretion of proangiogenic and anti-inflammatory cytokines, which in turn reduced inflammation and fibrosis in the MI rats. Consequently, a vascular niche was created in the ischemic heart that aided in myocardial repair by improving MSC survival, differentiation, and vessel specification [155]. In another study, Wu et al. reported the use of MSCs overexpressing C-X-C chemokine receptor type 4 (CXCR4), a key regulator of progenitor cell function, to treat ischemic heart injury. Neonatal and adult cardiomyocytes were supplemented with conditioned media (CM) obtained from culturing plain MSCs (MSC^Null^), CXCR-4 overexpressed MSCs (MSC^CX4^), and MSCs transduced with CXCR-4 gene specific siRNA (MSC^siR^) under hypoxic conditions. This study observed that the use of MSC^CX4^ increased the proliferation of cardiomyocytes and enhanced the paracrine secretion of VEGF, TGF-β2, and cyclin 2. In contrast, the levels of these secretions were significantly lower in MSC^Null^ or MSC^siR^. The transplantation of these cells into the MI rat models resulted in an improved homing, vascular density and angiogenesis, and a reduced LV wall fibrosis with an overall enhanced cardiac function after MI [140]. In another study by Liang et al., the MSC^CX4^ was shown to induce higher VEGF-A and HIF-1α levels, in addition to promoting EC differentiation and new vessel formation. The in vivo effects of implanting cell patches of MSC^Null^ and MSC^CX4^ into rat models of MI enhanced the neovascularization of ischemic tissues, which communicated with the native coronary arteries [77]. Additionally, Li et al., employed anti-apoptotic Bcl-2 (B-cell leukemia/lymphoma 2 protein) gene modified MSCs for MI, whereby they observed that Bcl-2 protected the MSCs against apoptosis and boosted the VEGF secretion by a substantial fold in vitro under hypoxic conditions. In vivo, they observed cell survival and angiogenesis, with a reduced infarct size indicating their potential to promote functional recovery after MI [75]. Improved angiogenesis was also observed for MSCs transfected with farnesoid X receptor (FXR) [142], Wnt11  [72], and islet-1 (ISL1) [143].

On the contrary, there have been numerous attempts to investigate the dual expression of genes following ischemia. Shujia et al., for instance, investigated the angiomyogenic potential of rat BM-MSCs by co-overexpressing Akt and angiopoietin-1 (Ang-1). Rat models of acute MI were created by permanent occlusion of the left anterior descending coronary artery, and injections of DMEM without cells, MSCs without transduction, or MSCs transduced with both Akt and Ang-1 (MAA group) were administered intramyocardially. Even three months after transplantation, the blood vessel density owing to MAA group was found to be the highest in comparison to other groups, which also experienced the development of a smooth muscle covering over them. A long-term treatment for infarcted hearts was made possible by this research [113]. A detailed analysis of the studies that use eMSCs to therapeutically target cardiac ischemia has been presented in Table 1.

**Table 1:** Therapeutic applications of eMSCs against cardiac ischemia

Cell source	Indication	Gene of interest	Route of administration	Major therapeutic benefits	References
hUCB-MCs	Cardiovascular disease and diabetic cardiomyopathy	VEGF, FGF2, and SDF1α	Subcutaneous	• Enhanced blood vessel formation in vivo	[31]
rBM-MSCs	Myocardial infarction	VEGF	Intramyocardial	• Promote endothelial proliferation in vitro • Improved transplanted MSC survival, neovascularization and preserved heart function in vivo	[157]
rBM-MSCs	Myocardial infarction	VEGF	Intramyocardial	• Enhanced capillary formation in the infarcts, and LV remodelling	[94]
hUC-MSCs	Myocardial infarction	HGF	Intramuscular	• Reduced apoptosis, enhanced paracrine secretion, increased angiogenesis and enhanced cardiomyocyte proliferation	[154]
hBM-MSCs	Myocardial infarction	HGF	Intramyocardial	• Improved LV function, reduced scar area and enhanced angiogenesis	[135]
pBM-MSCs	Myocardial infarction	HIF1α	Endomyocardial	• Increased angiogenesis and cardiac function, supported by reduced infarct size	[30]
hUC-MSCs	Myocardial infarction	eNOS	Intramyocardial	• Improved vascularization, and cardiac function with reduced infarct size	[18]
hBM-MSCs	Myocardial infarction	ILK	Intramuscular	• Improved angiogenesis, better cardiac remodelling and reduced infarct and fibrosis	[150]
mBM-MSCs	Myocardial infarction	MiR-126	Intramuscular	• Stimulation of vasculogenesis via the AKT/ERK – related pathway	[15]
rBM-MSCs	Myocardial ischemia	HO-1	Intramyocardial	• Increased capillary density, reduced apoptosis, and infarct area	[128]
rBM-MSCs	Myocardial infarction	SDF-1α	Intramyocardial	• Increased capillary density, thicker left ventricular (LV) wall, and smaller infarcts and fibrosis	[123]
mBM-MSCs	Myocardial infarction	GSK-3β	Intraperitoneal	• Improved paracrine function and capillary density, enhanced LV function	GSK-3β
rBM-MNCs	Myocardial infarction	FoxC1	Intramyocardial	• Reduced inflammation and fibrosis, with improved paracrine function	[155]
rBM-MSCs	Ischemic heart injury	CXCR4	Intravenous	• Increased cardiomyocyte proliferation and paracrine secretion, improved homing, and angiogenesis, and reduced LV wall fibrosis	[140]
rBM-MSCs	Myocardial infarction	CXCR4	Peritoneal cell patch	• Enhanced vessel formation, endothelial differentiation in vitro • Reduced infarct, restored cardiac function, enhanced neovascularization	[77]
rBM-MSCs	Myocardial infarction	Bcl-2	Intracardiac	• Anti-apoptotic, increased VEGF secretion in vitro under hypoxia • In vivo cell survival, increased angiogenesis and functional recovery	[75]
mAD-MSCs	Myocardial infarction	FXR	Intramyocardial	• Improved survival and paracrine secretion of cells with enhanced cardioprotection	[142]
rAD-MSCs	Hypoxic injury	Wnt11	-	• Enhanced cardiomyocyte protection, preserved mitochondrial membrane integrity, and enhanced paracrine function	[72]
hMSCs	Myocardial infarction	ISL1	Intramuscular	• Promote cell survival, boost paracrine activity and cardioprotection	[143]
rBM-MSCs	Myocardial infarction	Akt and Ang-1	Intramyocardial	• Enhanced blood vessel density and angiomyogenic response	[113]

UCB-MCs—Umbilical cord blood mononuclear cells, BM-MNCs – Bone marrow derived mononuclear cells, BM-MSC (Bone marrow derived mesenchymal stromal cells), AD-MSC (Adipose tissue derived mesenchymal stromal cells), UC-MSC (Umbilical cord derived mesenchymal stromal cells), LV – left ventricular, VEGF – vascular endothelial growth factor, FGF2 – fibroblast growth factor 2, SDF1α – stromal cell derived factor 1α, HGF – hepatocyte growth factor, HIF1α—hypoxia-induced factor 1α, eNOS—endothelial nitric oxide synthases, ILK—integrin-linked kinase, MiR-126 – miRNA 126; HO-1 – heme oxygenase 1, GSK-3β—glycogen synthase kinase-3β, FoxC1—Forkhead box C1, CXCR4—C-X-C chemokine receptor type 4, Bcl-2—B-cell leukemia/lymphoma 2 protein, FXR—farnesoid X receptor, ISL1—islet-1, Ang-1—angiopoietin-1

### Cerebral Ischemia

Stroke is the second largest cause of death globally and causes an elevated risk of disability [131]. Likewise, ischemic stroke, also known as cerebral ischemia, is one of the most prevalent forms of stroke. It is a cerebrovascular illness that is defined by a disruption in blood flow to the brain, which results in brain infarction, dysfunction, and neuronal damage [42, 70, 73]. Proinflammatory cytokines are released when oxidative stress activates macrophages in the ischemic area, impairing the microenvironment of the brain through inflammation [4, 50]. Current therapies such as tissue plasminogen activator therapy-induced thrombolysis and endovascular canalization, although considered the accepted standard of care, often have a limited therapeutic window and cause lifelong disabilities [42, 70, 73]. However, a second therapeutic window that begins a few days after the stroke is available for nerve regeneration. This is an opportunity to use stem cells because they can differentiate and regenerate damaged tissues [42, 71].. Numerous studies have shown that the injection of human MSCs into rat models of cerebral ischemia leads to angiogenesis and functional recovery [16, 17, 51, 91]. It takes at least 3 to 4 days for MSCs transplanted into ischemic brain areas to begin secreting angiogenic factors, which by that time may have caused neuronal degradation [64]. Therefore, the present focus is to modify the MSCs to overcome this barrier.

There have been limited studies on the use of angiogenesis factor overexpressed MSCs for cerebral ischemia. A study by Lai et al., employed VEGF gene overexpressed MSCs in a rat model of cerebral infarction created through middle cerebral artery occlusion (MCAO) infarction/reperfusion (I/R). Following transfection, these VEGF-MSCs displayed 28% increase in VEGF concentration. Compared to using normal MSCs, they noticed an increase in the levels of VEGF and Angiopoetin-2 (Ang-2) surrounding the infarct after I.V. transplantation of VEGF-MSCs into the model. Together, these two proteins caused angiogenesis, which restored blood flow to the areas surrounding the cerebral infarction and resulted in the recovery of neurological function [64]. Toyama et al. treated cerebral ischemia by using a dual gene overexpressed strategy. The function of the adult BM-MSCs that had been transfected with both the VEGF and Ang-1 (Ang-VEGF-MSC) genes was compared to that of the MSCs that had been transfected with individual genes (Ang-MSC and VEGF-MSC), with normal BM-MSCs acting as the control. While transplantation of VEGF-MSCs alone led to worsened functional outcome, the other 3 group effectively reduced the infarct size and induced angiogenesis, with the highest functional recovery being shown by Ang-VEGF-MSCs. This approach sheds light on maximising angiogenesis for beneficial cerebral ischemia therapies [127]. Another factor that is known to induce revascularization in the ischemic lesions of cerebral ischemia is hypoxia-inducible factor 1α (HIF1α) [145]. In rat models of cerebral ischemia, Yang et al. injected rat BM-MSCs that overexpressed mutant HIF1α. To control HIF1α expression, the gene construct included mutations of proline at position 564 and asparagine at position 803 respectively. The VEGF protein levels and microvessel density estimated at day 7, 14 and 28 showed seemed to be higher in the animals treated with overexpressed cells that caused revascularization. This was accompanied by reduced infarct size and motor function recovery, providing a promising therapy for cerebral ischemia [145].

Onda et al. also produced Ang-1 transfected adult BM-MSCs to investigate their impact on cerebral ischemia in contrast to naïve MSCs. After receiving an I.V. infusion of Ang-1-MSCs and naïve MSCs, the MCAO rat models exhibited improved angiogenesis, particularly at the ischemic lesion's edge, where there was an increase in blood flow and neovascularization. They also led to the tissue's functional recovery and a decrease in the lesion's volume. The Ang-1-MSC group exhibited a greater degree of all these effects, indicating their superior angiogenic capacity over naïve MSCs [98]. FGF-1 (fibroblast growth factor-1) is another crucial factor that is known to be angiogenic [132] and promotes proliferation and differentiation of neuronal cells [107]. Hoseini et al., developed FGF-1 gene-engineered rat AD-MSCs that showed to support fibroblast proliferation and tube formation in HUVECs [44]. In addition to their angiogenic potential in vitro, they also showed elevated FGF-1 level in the ischemic hemispheres, reduced infarct volume and apoptosis, and significantly recovered the neurological function [33]. Similarly, PlGF-overexpressed MSCs also exhibited significant amount of angiogenesis, neuroprotection, and functional restoration following cerebral ischemia [81]. Thrombospondin-4 (TSP4) is an endothelial regenerative marker, that when overexpressed in BM-MSCs, resulted in improved angiogenesis in vitro, and notable improvements in the neurological functions and vascular recovery post infusion into rat MCAO models [153]. This was mediated by the TGF-β/Smad2/3 signaling pathway in HUVECs, which is crucial for mediating angiogenic responses suggesting that the therapeutic effect was mainly from the paracrine signaling mechanisms.

In the cerebral ischemia models, overexpression of a few other genes in MSCs have also been examined for promoting angiogenesis. One neurotrophic factor produced by MSCs that is important for neurogenesis is brain-derived neurotrophic factor (BDNF) [20]. In a study, a rat model of acute ischemic stroke was implanted with MSCs that had been genetically modified to overexpress BDNF. These cells were delivered using hydrogel to ensure prolonged survival. Seven days after transplantation, elevated VEGF and IGF-1 levels were observed, and fourteen days later, the induction of angiogenesis was detected using CD-31 expression [133]. Another gene necessary for both cell delivery into the brain and neural repair following a stroke is C–C motif chemokine ligand 2 (CCL2) [2, 105]. The CCL2-engineered MSC-treated acute stroke rats showed higher CCL2 levels and enhanced stem cell migration into the ischemic regions. Furthermore, increased neurogenesis and angiogenesis led to endogenous brain repair. Additionally, a decrease in the infarct volume and decreased inflammation were noted.[68]. An overview of the current research on the role of eMSCs against cerebral ischemia has been presented in Table 2.

**Table 2:** Therapeutic applications of eMSCs against cerebral ischemia

Cell source	Indication	Gene of interest	Route of administration	Major therapeutic benefits	References
rBM-MSCs	Cerebral infarction	VEGF	Intravenous	• Increased VEGF and Ang-1 levels, restoration of blood flow and neural function	[64]
hBM-MSCs	Cerebral ischemia	VEGF and Ang-1	Intravenous	• Reduced the infarct size and induced angiogenesis	[127]
rBM-MSCs	Cerebral ischemia	HIF1α	Intravenous	• Increased VEGF protein levels and enhanced microvessel density, reduced infarct size and functional recovery	[145]
hBM-MSCs	Cerebral ischemia	Ang-1	Intravenous	• Improved angiogenesis and neovascularization, functional recovery and decrease in lesion volume	[98]
rAD-MSCs		FGF-1		• Support fibroblast proliferation and tube formation in vitro	[44]
rAD-MSC	Ischemic stroke	FGF-1	Intravenous	• Improved neural function, and reduced apoptosis and infarct volume	[33]
hBM-MSCs	Cerebral ischemia	PlGF	Intravenous	• Improved angiogenesis, neuroprotection, and functional restoration	[81]
rBM-MSCs	Cerebral ischemia	TSP4	Intravenous	• Improved angiogenesis in vitro through TGF-β/Smad2/3 pathway, and neurological function and vascular recovery	[153]
mBM-MSC	Acute ischemic stroke	BDNF	Trans-septal approach	• Elevated VEGF and IGF-1 levels and induction of angiogenesis	[133]
hUC-MSCs	Acute ischemic stroke	CCL2	Intravenous	• Functional recovery with reduced stroke volume, increased angiogenesis and endogenous neurogenesis, and decreased neuro-inflammation	[68]

BM-MSCs (Bone marrow derived mesenchymal stromal cells), AD-MSCs (Adipose tissue derived mesenchymal stromal cells), UC-MSCs (Umbilical cord derived mesenchymal stromal cells), VEGF – vascular endothelial growth factor, Ang-1 – angiopoietin 1, HIF1α—hypoxia-induced factor 1α, FGF-1—fibroblast growth factor 1, PlGF – placental growth factor, TSP4 – thrombospondin 4, BDNF—brain-derived neurotrophic factor, CCL2—C–C motif chemokine ligand 2, IGF-1—Insulin-like growth factor 1

## Challenges of Using eMSCs

Due to their entrapment in the lymphoid or lung systems, MSC transplantation alone is susceptible to several difficulties, including their restricted capacity for homing and survival in injured tissue [95, 139]). Be it unmodified MSCs or ones that have undergone gene modification, the mode of administration plays a crucial role in determining the outcome of the therapy. This is explained by the challenging microenvironment that the cells experience during ischemia, which may reduce the effectiveness of their treatment. Studies reveal that although I.V. administration is safer but less effective in reaching brain tissue, intraarterial (I.A.) delivery can effectively target the brain, but it carries a higher risk of cerebral lesions and does not improve functional recovery [3]. In the context of cardiac ischemia, modified mRNA-based gene delivery has demonstrated promise owing to its high potency and efficiency, indicating that the mode of administration can have a major impact on therapeutic efficacy [149]. While intramyocardial routes are direct, they are invasive and restricts the possibility of multiple interventions [103]. As a result, there are a number of grave concerns regarding the use of eMSCs in clinical settings. Even though many of the studies included in this review have produced encouraging results, they continue to face a few barriers, the most significant of which are insertional mutagenesis and malignant transformation [38]. Another factor that restricts the use of viral vectors for genetic modification is the safety risk involved with the vector selection [95]. On the other hand, non-viral vectors may reduce the transfection efficiency and, thus, the therapeutic potential of eMSCs. Thus, one of the key variables influencing the in vivo outcomes is the appropriate vector selection [138]. Another factor that is vital to the maintenance of these cells is the culture conditions that effectively sustain the modified cells. Furthermore, the process of isolating cells, the number of cells administered, the administration route, and the injection site are all legitimate points that need to be optimized for applications [139]. The source of MSCs is also vital as their properties and culture conditions differ with respect to the tissue from which it was isolated. Studies show that adult bone marrow (BM)-derived MSCs, which are the gold standard, differ from perinatal tissues like umbilical cord (UC) and amniotic membrane (AM) MSCs in their properties [137]. Although UC and AM MSCs are similar, AM-MSCs show more variability between donors, suggesting that donor-specific factors influence AM-MSCs more than they do to UC-MSCs [137]. Furthermore, the inherent variability in MSC-based therapies is exacerbated by the distinct gene expression profiles and functional characteristics displayed by MSCs from different tissues [11]. In addition, the most crucial issue that still needs to be resolved is the loss of transplanted cells and the long-term fate of the cells. There is also a shortage of clinical evidence to back up the effectiveness and safety of eMSCs. The answers to these questions will determine the fate of gene modified MSCs.

Apart from safety concerns with the choice of vector, the use of eMSCs for therapeutic angiogenesis in cardiac and cerebral angiogenesis also presents certain limitations. Despite genetic modification to enhance their properties, MSCs' survival and therapeutic efficacy are significantly reduced by the harsh microenvironment of ischemic tissues  [14, 53]. The long culture times required before transplantation further limit the effectiveness of MSCs and may increase senescence and reduce functionality [156]. While genetic modifications aim to improve characteristics such as paracrine signaling and migration, their engraftment and retention in target tissues remain inadequate, which further undermines their potential benefits [92]. Finally, the complexity of the immune response and the variation in the biological behavior of modified MSCs can lead to unexpected outcomes in clinical settings [53].

Although more than a thousand MSC-based clinical trials have been registered, their clinical approval is hampered by a lack of knowledge about MSC biology, specifically with regard to their in vivo identity and mechanism [56]. Additionally, while preconditioning techniques and genetic alterations have demonstrated promise in boosting MSCs' therapeutic potential, consistently producing positive outcomes in clinical trials is still a significant challenge [87, 89]. Finally, the lack of efficiency in the production of MSC-derived exosomes, which is essential for their paracrine effects, restricts their use in regenerative medicine [99]. The successful clinical translation of gene-modified MSC therapies depends on addressing these obstacles. Thus, major challenges remain to be resolved in order to optimize genetically modified MSC therapies for ischemic conditions, despite recent progress.

## Conclusion and Future Perspectives

The poor survival rate following transplantation limits the therapeutic efficacy of MSC transplantation, which in turn restricts their use. This review explores the idea of strengthening the MSCs therapeutic ability by engineering the cells with growth factors, cytokines, and proteins. Several preclinical studies supporting the application of eMSCs in cardiac and brain ischemia have been included in this review. Enhancing vascularization in ischemic lesions using growth factor overexpressed MSCs is the main therapeutic approach used in these cases. The enhanced paracrine secretion, differentiation into ECs, reduced apoptosis, and shrinking of the infarction volume were the common effects observed in most of the studies. Nevertheless, there are certain safety issues related to eMSCs that must be taken care of.

When compared to conventional therapies, genetically modified MSCs provide notable advantages in the treatment of ischemic conditions. MSCs can enhance the effectiveness of traditional revascularization therapies by fostering an environment that is conducive to the regeneration and repair of ischemic lesions, as opposed to immediate restoration of blood flow that is critical in acute ischemic events. In addition to the targeted approach of gene modification, homing to the site of injury, anti-inflammatory, and paracrine signaling are the beneficial properties that make engineered MSC-based treatments superior to other therapies. MSC-derived EVs are a promising new field that has the notable advantage of being less immunogenic due to their cell-free nature. Nonetheless, further research in required to standardize the isolation procedures and determine their therapeutic effect in vivo.Although most of the studies in this review highlight the progress made in the field of engineering MSCs for therapeutic angiogenesis, more preclinical and clinical data would be beneficial to support these developments. There are several safety concerns that arise with both viral and non-viral vector mediated transfections that must be fully addressed. Optimising the primary culture conditions, selecting the MSCs' source, dosage, and vectors, route of administration, among other factors, may result in higher transfection efficiencies [138, 141]. Clinical trials are going to be one of the most crucial factors in determining whether eMSCs can be translated into useful applications in the future.

## Data Availability

Not applicable.
